# *Vitis* Phylogenomics: Hybridization Intensities from a SNP Array Outperform Genotype Calls

**DOI:** 10.1371/journal.pone.0078680

**Published:** 2013-11-13

**Authors:** Allison J. Miller, Naim Matasci, Heidi Schwaninger, Mallikarjuna K. Aradhya, Bernard Prins, Gan-Yuan Zhong, Charles Simon, Edward S. Buckler, Sean Myles

**Affiliations:** 1 Department of Biology, Saint Louis University, St. Louis, Missouri, United States of America; 2 Department of Ecology and Evolutionary Biology and iPlant Collaborative, University of Arizona, Tucson, Arizona, United States of America; 3 United States Department of Agriculture, Agricultural Research Service, Plant Genetic Resources Unit, New York State Agricultural Experiment Station, Cornell University, Geneva, New York, United States of America; 4 United States Department of Agriculture, Agricultural Research Service, National Clonal Germplasm Repository, University of California Davis, Davis, California, United States of America; 5 United States Department of Agriculture, Agricultural Research Service, Grape Genetic Research Unit, New York Agricultural Experiment Station, Cornell University, Geneva, New York, United States of America; 6 Institute for Genomic Diversity, Cornell University, Ithaca, New York, United States of America; 7 Faculty of Agriculture, Dalhousie University, Truro, Canada; Wuhan Botanical Garden, Chinese Academy of Sciences, Wuhan, China

## Abstract

Understanding relationships among species is a fundamental goal of evolutionary biology. Single nucleotide polymorphisms (SNPs) identified through next generation sequencing and related technologies enable phylogeny reconstruction by providing unprecedented numbers of characters for analysis. One approach to SNP-based phylogeny reconstruction is to identify SNPs in a subset of individuals, and then to compile SNPs on an array that can be used to genotype additional samples at hundreds or thousands of sites simultaneously. Although powerful and efficient, this method is subject to ascertainment bias because applying variation discovered in a representative subset to a larger sample favors identification of SNPs with high minor allele frequencies and introduces bias against rare alleles. Here, we demonstrate that the use of hybridization intensity data, rather than genotype calls, reduces the effects of ascertainment bias. Whereas traditional SNP calls assess known variants based on diversity housed in the discovery panel, hybridization intensity data survey variation in the broader sample pool, regardless of whether those variants are present in the initial SNP discovery process. We apply SNP genotype and hybridization intensity data derived from the Vitis9kSNP array developed for grape to show the effects of ascertainment bias and to reconstruct evolutionary relationships among *Vitis* species. We demonstrate that phylogenies constructed using hybridization intensities suffer less from the distorting effects of ascertainment bias, and are thus more accurate than phylogenies based on genotype calls. Moreover, we reconstruct the phylogeny of the genus *Vitis* using hybridization data, show that North American subgenus *Vitis* species are monophyletic, and resolve several previously poorly known relationships among North American species. This study builds on earlier work that applied the Vitis9kSNP array to evolutionary questions within *Vitis vinifera* and has general implications for addressing ascertainment bias in array-enabled phylogeny reconstruction.

## Introduction

Understanding relationships among species is the basis for modern classification schemes and provides the requisite framework for ecological and evolutionary analyses of diversity patterns and diversification processes [1], [2]. Large-scale coordinated research programs, together with technical and analytical advances, have facilitated significant progress in current understanding of organismal phylogeny. Despite this, uncertainty regarding evolutionary relationships among species remains in many groups, including several that include economically important species such as apples [3], [4], grapes [5]–[7], potatoes [8], and wheat [9].

Over the past five years, nearly all sub-disciplines within biology have been revolutionized in the wake of the genomics era [10], [11]. Widespread adoption of next-generation sequencing (NGS) technologies have reduced the cost of DNA sequencing by orders of magnitude providing unprecedented access to the genome of an organism (www.genome.gov/sequencingcosts). One application of NGS is single nucleotide polymorphism (SNP) discovery through whole-genome sequencing or comparative sequence analysis of expressed sequence tags (ESTs) or reduced-representation libraries (RRLs) [12]–[14]. Resulting SNPs can be used to construct a SNP array, a compilation of hundreds, thousands, or even millions of polymorphic sites that enables genotyping of an individual at multiple loci simultaneously (e.g., [13]). To date, SNP arrays have been developed primarily in systems for which large amounts of genomic data are already available, including model organisms with sequenced genomes or domesticated species with significant EST libraries [15]–[19]. In combination with phenotypic data, SNP arrays have been used extensively in linkage mapping (e.g., [20]), association genetics (e.g., [21]), and genome-wide association studies [22], [23] and have been particularly useful in screening variation in crop species [24]. High-throughput genotyping via SNP arrays has contributed to current understanding of the genetic basis of agriculturally important traits and is supporting crop improvement efforts by accelerating marker-assisted selection and genomic selection [25].

In addition to crop improvement, SNP microarray technology holds great promise for studying evolutionary processes that shape variation in natural populations [26]–[30]. For example, SNP arrays have been used to characterize the genetic basis of local adaptation in *Arabidopsis* [31] [32], Douglas fir [33], loblolly pine [34], poplar [35], and Sitka Spruce [36], among others. The convenience of genotyping thousands of sites at the same time, together with the economy of scale, has propelled the use of array-generated genotypic data in a variety of evolutionary questions.

Phylogeny reconstruction based on genome-wide data (“phylogenomics”) is an exciting and important development in evolutionary biology [10], [37]. SNP arrays present a potentially valuable source of data for this purpose and have already been used to genotype large numbers of individuals across multiple species. For example, evolutionary relationships among higher ruminants (e.g., cattle, sheep, goats, antelopes, deer, giraffes, pronghorn) were estimated using the Bovine SNP50 BeadChip, an array developed from variation detected among six cattle breeds and from heterozygous sites in the sequenced cattle genome [38], [39]. Phylogenomic analyses based on 678 animals representing 61 species genotyped at more than 40,000 SNP sites yielded support for established clades and identified several new relationships. Similar studies have been completed in humans [16], horses and their wild relatives [13], and old world monkeys [40].

Utilization of SNP arrays involves applying variation discovered in one or a few individuals to a large range of accessions [41], [42]. The number and diversity of individuals used in the SNP discovery process (the discovery panel) almost always leads to some degree of ascertainment bias because the discovery panel consists of only a small subset of the individuals to be genotyped on the array [43], [44]. Frequently, the discovery panel favors identification of SNPs with high minor allele frequencies, introducing bias against rare alleles [45]. Ascertainment bias becomes particularly acute when SNPs identified for one level of analysis (e.g., within species comparisons) are used at different scales (e.g., among species comparisons, as in phylogeny reconstruction) [30], [46]. Indeed, it has been shown that the application of SNPs identified in a discovery panel to a broad set of samples is accompanied by losses in utility, particularly as genotyping is attempted for individuals that are increasingly evolutionarily divergent from the panel accessions [47]–[51]. We expect ascertainment bias to be particularly severe when assaying variation across a highly diverse genus like *Vitis*, where common ancestry between species is expected to date back tens of millions of years [5], [7].

Several approaches to reduce the effects of ascertainment bias have been proposed (reviewed in [45], [46], one of which involves the use of hybridization intensity data rather than genotype calls. Hybridization intensity data capture otherwise undetectable variation in SNP array data known as “off-target variants”, variation in genomic DNA that differs from the expected variant targeted by the array design [49]. Characterizing site variation without directly querying alternative alleles at a locus has been used to identify polymorphisms between maize inbred lines [52], [53], in association mapping in *Arabidopsis* [54], and in phylogeny reconstruction [49]. Summary statistics of fluorescence intensity values have been shown to outperform bi-allelic genotype calls for the purposes of linkage mapping in grape (Myles et al. unpublished data). Whereas traditional SNP calls assess known variants based on diversity housed in the discovery panel, hybridization intensity data characterize variation in the broader sample pool, regardless of whether or not those variants are present in the individuals used in the initial SNP discovery process.

In this study, we apply SNP genotype and hybridization intensity data derived from the Vitis9kSNP array developed for grape [14], [55] to characterize the effects of ascertainment bias and to reconstruct evolutionary relationships among *Vitis* species. A North Temperate genus comprising approximately 60 species, *Vitis* includes at least 14 species and three named hybrid taxa native to North America, one species complex in Europe (the cultivated grape *V. vinifera* ssp. *vinifera* (“vinifera”) and its wild progenitor *V. vinifera* ssp. *sylvestris* (“sylvestris”) [55]–[57], and 37 species in China [58], [59]. Previous phylogenetic analyses have demonstrated that *Vitis* is monophyletic and consists of two subgenera, subgenus *Muscadinia* (N = 2–3 North American species) and subgenus *Vitis* (N = ∼60 species found in North America, Europe, and Asia) [5]–[7], [55], [56], [59]–[63]. To date, chloroplast and nuclear sequence data, amplified fragment length polymorphism (AFLP), and microsatellites have been employed to describe the evolutionary relationships among subgenus *Vitis* species [5]–[7], [60], [63]; these studies have generated support for some relationships within the genus, but several questions remain. Most notably, it is unclear if the North American subgenus *Vitis* species are monophyletic, and species-level relationships within the North American clades of subgenus *Vitis* remain largely unresolved.

*Vitis* presents an ideal system in which to explore the utility of SNP array data for phylogenetic analysis and to assess the effects of ascertainment bias on phylogeny reconstruction. This study system exhibits many attributes believed to exacerbate ascertainment bias: 1) *Vitis* is highly heterozygous; 2) common ancestry between species dates to at least 10 million years ago [5], [7]; and 3) the Vitis9kSNP array discovery panel was built using 17 individuals (eleven *V. vinifera* cultivars, one individual each of *V. amurensis, V. cinerea, V. labrusca, V. palmata, V. rotundifolia*, and *V. vinifera* ssp. *sylvestris*) but has been used to survey larger numbers of samples from a variety of taxa. In addition, previous phylogenetic analyses of *Vitis* have demonstrated consistent support for some relationships, for example, the progenitor-descendant relationship between *sylvestris* and the cultivated grape *vinifera*. Clades like this present an opportunity to evaluate whether genotype data or hybridization intensity data (or both) have the capacity to recover known relationships.

Here, we use the Vitis9kSNP array to characterize variation in approximately one third of *Vitis* species, genotyping over 1100 accessions at nearly 9000 sites [14]. We demonstrate that phylogenies constructed using hybridization intensities suffer less from the distorting effects of ascertainment bias, and are thus more accurate, than phylogenies based on genotype calls. Moreover, we reconstruct the phylogeny of the genus *Vitis* using hybridization data, provide evidence to suggest that North American subgenus *Vitis* species are monophyletic, and identify several species-level relationships among North American *Vitis* species. This study builds on previous work that applied the Vitis9kSNP array to evolutionary questions within *Vitis* [55]; Myles et al. unpublished data), and has general implications for addressing ascertainment bias in array-enabled phylogeny reconstruction.

## Methods

### Sampling

Leaves for DNA extraction were collected from the USDA grape germplasm collections in Davis, California, and Geneva, New York. Permission for tissue collection was obtained from the local USDA authorities. DNA was extracted using DNeasy Plant Mini Kits (Qiagen) and 1173 accessions representing 19 taxa (16 unique species, two hybrid taxa, one species with two intra-specific groups) were genotyped using the Vitis9kSNP array, which includes 8898 SNPs [14], [55] (Table 1).

**Table 1:** Accessions used in the SNP Analyses.

Subgenus	Geographic area	Species	N = 1173 (before filters)	N = 1030 (after filters)	Common name
Subg. Muscadinia	North America	*V. rotundifolia* Michaux	71	68	Muscadine grape
Subg. Vitis	North America	*Vitis acerifolia* Rafinesque	18	15	Bush grape
Subg. Vitis	North America	*V. aestivalis* Michaux	55	42	Summer grape
Subg. Vitis	North America	*V. cinerea* (Engelm). Engelm. Ex Millardet	98	70	Downy grape, sweet winter grape, graybark grape
Subg. Vitis	North America	*V. doaniana*	5		
Subg. Vitis	North America	*V. girdiana* Munson	4	4	Desert wild grape
Subg. Vitis	North America	*V. labrusca* Linneaus	36	23	Fox grape
Subg. Vitis	North America; limestone hills on the Edwards Plateau	*V. monticola* Buckley	5	4	Sweet mountain grape
Subg. Vitis	North America (Texas), Mexico	*V. mustangensis* Buckley	7	5	Mustang grape
Subg. Vitis	North America	*V. palmata* Vahl.	13	9	Catbird grape, Red grape
Subg. Vitis	North America	*V. riparia* Michaux	113	73	Riverbank grape, frost grape
Subg. Vitis	North America	*V. rupestris* Scheele	48	36	Rock grape, sand grape
Subg. Vitis	North America	*V. vulpina* L.	23	15	Winter grape
Subg. Vitis	North America (Texas)	*V. x champinii* Planch. ( = *V. mustangensis* and *V. rupestris*	15	12	Champin's grape
Subg. Vitis	Eurasia (China, Japan, Russia)	*V. amurensis* Rupr.	22	13	
Subg. Vitis	East Asia (China, Japan)	*V. coignetiae* Pulliat ex Planch.	6	3	
Subg. Vitis	East Asia (China)	*V. piasezkii* Maxm. *pagnuccii* (Rom. Caill.) Rehder	9	9	
Subg. Vitis	Eurasia	*V. sylvestris* W. Bartram ( = *V. vinifera* subsp. *sylvestris* (C. C. Gmel.) Hegi	59	59	European grape
Subg. Vitis	Eurasia	*V. vinifera* L. ( = V. vinifera subsp. vinifera)	570	570	

### Genotype data curation

An initial principal components analysis (PCA) was conducted in R using the genotype calls from the Vitis9kSNP array in order to examine whether or not individuals clustered according to their assigned species. SNPs with low genotype quality scores (GenCall<0.2), low SNP quality scores (GenTrain score<0.3), MAF<0.05 and >20% missing data were excluded, which resulted in a data set of 4073 SNPs. For PCA, SNPs were pruned for linkage disequilibrium (LD) using PLINK [64] by considering a window of 10 SNPs, removing one of a pair of SNPs if LD>0.5, and then shifting the window by three SNPs and repeating the procedure (plink command: –indep-pairwise 10 3 0.5). After these filters, 3231 SNPs remained for PCA. PCA was run and individuals representing obvious curation errors (i.e. those carrying one species name but obviously clustering with individuals from another species) were removed from the remaining analyses. After these data curation steps, 1030 samples remained from 18 different taxa. Genotype and intensity data are available in the dryad digital repository.

### Analyses of genotype data

To facilitate direct comparison between genotype data and hybridization intensity data, genotypes were used to calculate *F_ST_* among species. Only SNPs with MAF>0.05 and <20% missing data were included, resulting in 4073 SNPs and 1030 samples. We calculated a weighted average *F_ST_* between all pairs of species following equation 10 in [65]. The resulting *F_ST_* distance matrix was visualized with a multi-dimensional scaling (MDS) plot. The *F_ST_* distance matrix was then used to construct phylogenetic trees using the “nj” function in the ape package in R [66]. Neighbour-joining (NJ) trees rooted with *V. rotundifolia*, a representative of subgenus *Muscadinia*, were generated. To assess the impact of *V. vinifera* and *V. sylvestris* on the analysis, phylogenetic trees were constructed for the full dataset, as well as a reduced dataset with *V. vinifera* and *V. sylvestris* removed.

### Analyses of hybridization intensity data

We investigated whether the effects of ascertainment bias on phylogenetic structure could be circumvented using normalized intensity data. Instead of forcing the intensity values generated from the probes on the array into categorical variables, i.e. genotype calls, we used the normalized intensity values as “quantitative genotypes” and calculated genetic distances between species using these scores. To explore the utility of hybridization intensity data in the reconstruction of evolutionary relationships, normalized intensity data from all 8898 SNPs assayed by the Vitis9kSNP array were used to calculate a genetic distance matrix between species. This matrix was generated using the same set of samples and has the same format as the *F_ST_* distance matrix, facilitating comparison between relationships resolved using data from SNP genotype calls (previous section) and those resolved using the intensity data. For each SNP, the intensity data from the array consist of a normalized intensity for allele A (X) and a normalized intensity for allele B (Y) that captures information from an average of 30 probes querying that particular SNP. We investigated several summary statistics of these intensity values including X, Y, X+Y, X/(X+Y), ln(X/(X+Y)), ln(Y/(X+Y)), and ln(X/Y). To generate a single value for each SNP for each species, the median of the above summary statistics for each SNP was calculated for each species. Each of these matrices of summary statistics was converted into a distance matrix by calculating the Euclidean distances between each pair of species using the “dist” function in R. MDS plots were generated from these distance matrices to evaluate how well the intensity data captured relationships among samples. Distance matrices based on hybridization intensity were compared to one another and to the *F_ST_* distance matrix generated from the genotype calls using mantel tests with 10000 permutations. For each summary statistic described above, rooted trees (with *V. rotundifolia* as the root) were generated. Topologies of pairs of trees were compared using the method of [67] where the “distance” between two trees is defined as twice the number of internal branches defining different bipartitions of the tips.

## Results

### Assessment of curation error

**Figure 1:**
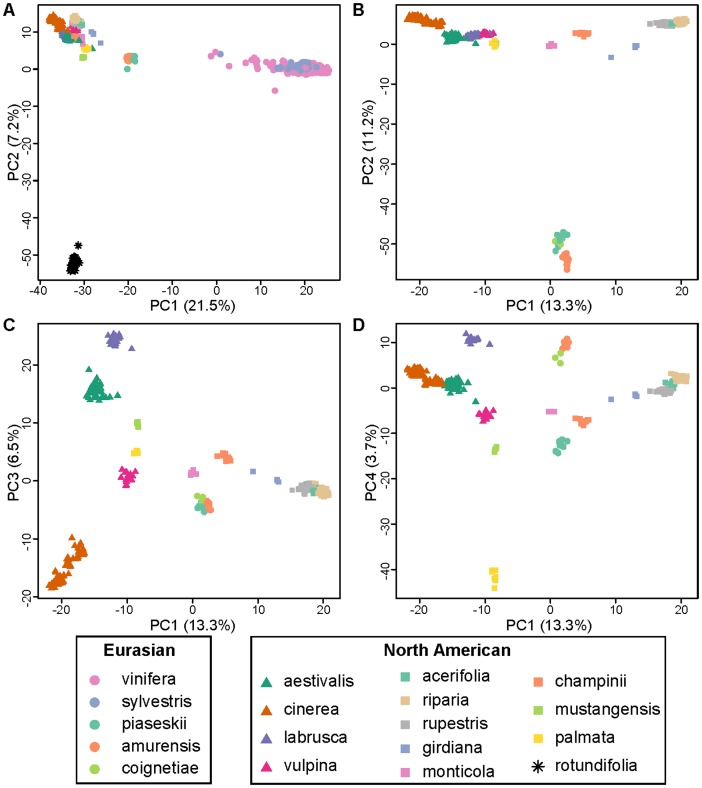
Principal components analysis (PCA) of 1030 *Vitis* samples using 3231 SNPs. The proportion of the variance explained is found within parentheses on each axis. **A) PCA with all samples included.** PC1 clearly separates *vinifera* and *sylvestris* from the other wild *Vitis* species while PC2 separates *rotundifolia* from all others. **B–D) PCA with** ***rotundifolia*** **,** ***sylvestris*** **and** ***vinifera*** **removed.** Examining the distances between individual samples in PC space confirms that the curated sample set used in the present study does not likely suffer from mislabeling or curation error that would lead to false phylogenetic inference.

Using PCA, we identified and removed 54 samples that clearly did not cluster according to their species membership, and likely represented curation errors in the collection. These samples represent approximately 5% of the genotyped samples from the USDA grape germplasm collection. Using PCA, we demonstrate that, after excluding these curation errors, the samples used in the present study indeed cluster according to their taxonomic identity (Fig. 1). The removal of *V. rotundifolia, V. sylvestris*, and *V. vinifera* (Figs. 1b–d) shows that, even for the North American and Eurasian species, sample mix up or curation errors are unlikely to contribute to false phylogenetic inferences.

### Assessment of ascertainment bias

**Figure 2:**
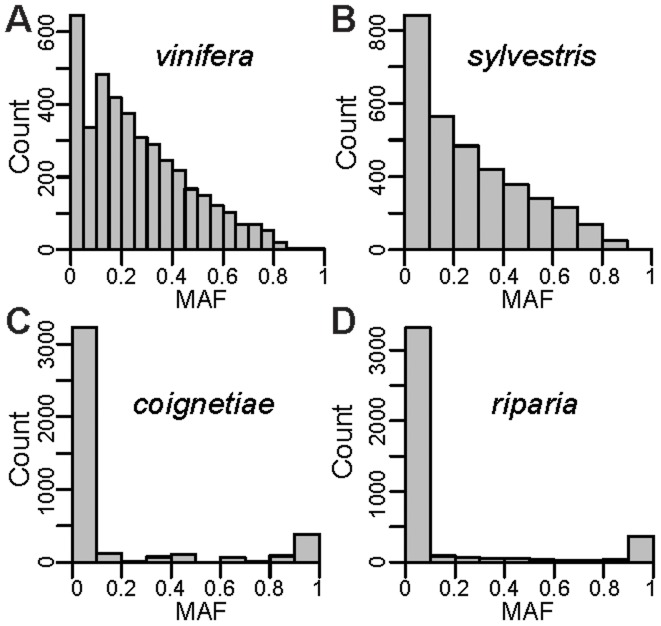
Minor allele frequency (MAF) for *vinifera* and *sylvestris* and two representative taxa, *V. coignetiae* from Asia and *V. riparia* from North America. MAF allele frequencies for other species look similar to *V. coignetiae* and *V. riparia* with a severe deficit of intermediate frequency alleles compared to *vinifera* and *sylvestris*.

**Figure 3:**
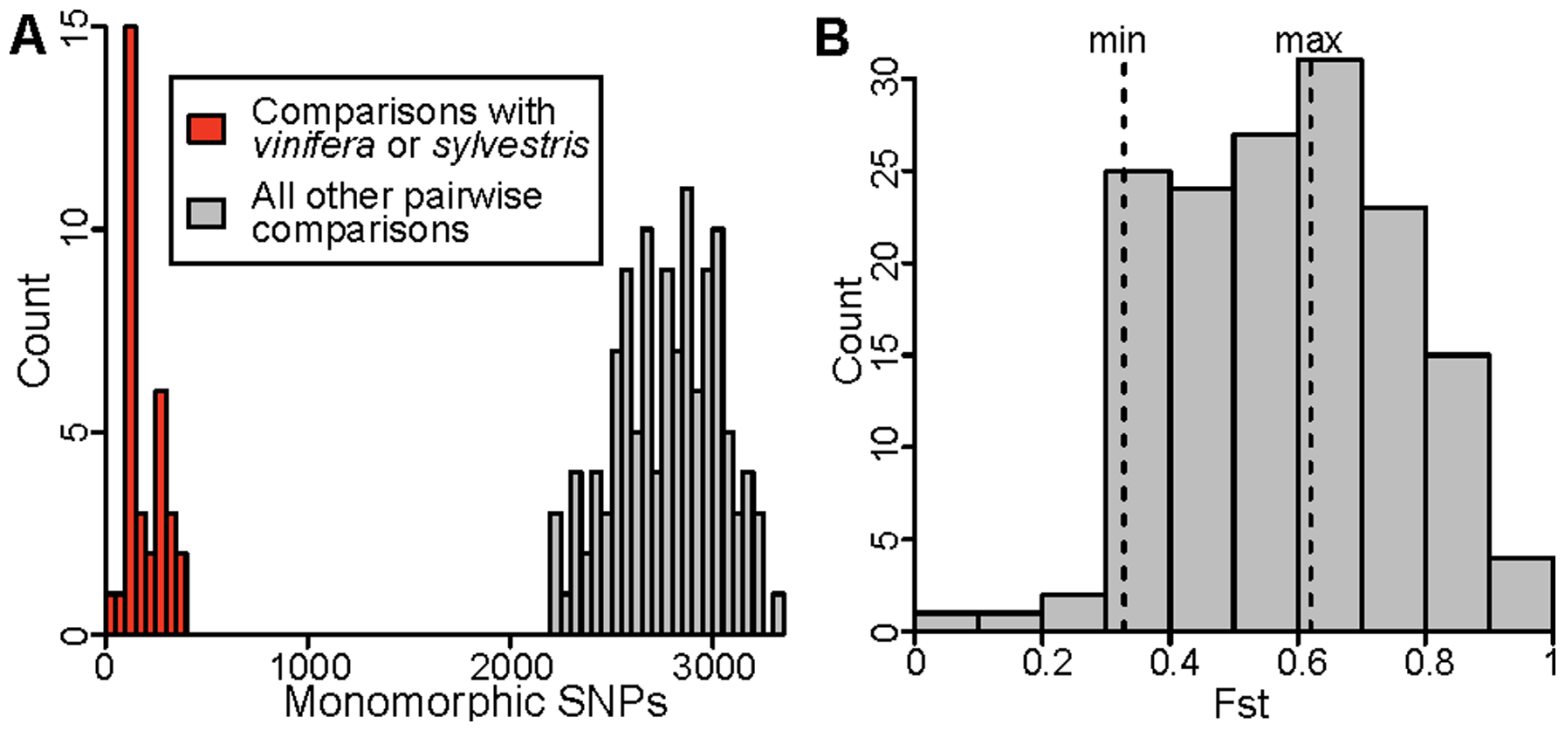
Evidence of ascertainment bias from the Vitis9KSNP array. **A**) Between-species comparisons with *vinifera* or *sylvestris* involve far fewer monomorphic SNPs than other comparisons. The number of monomorphic SNPs was calculated for every pairwise comparison between species. Because *vinifera* and *sylvestris* show an excess of intermediate frequency alleles compared to other *Vitis* species using the Vitis9KSNP array, comparisons involving *vinifera* or *sylvestris* display fewer monomorphic sites relative to comparisons involving other species pairs. **B**) The dotted lines indicated by “min” and “max” are the minimum and maximum *F_ST_* values from comparisons between *vinifera* or *sylvestris* and other species. The ascertainment bias results in intermediate *F_ST_* estimates with relatively little variation for pairwise comparisons between species involving *vinifera* or *sylvestris*.

The Vitis9kSNP array was constructed primarily to assay polymorphism within *V. vinifera*, with only a few probes designed specifically to query fixed differences among various *Vitis* species [14]. While the SNP data clearly group individuals according to their taxonomic identity (Fig. 1), we find pervasive evidence of ascertainment bias. For example, the minor allele frequency (MAF) distribution in *vinifera* and its closely related ancestor *sylvestris* shows a large excess of intermediate frequency alleles relative to other wild *Vitis* species examined (Fig. 2). This pattern of MAF distributions across species is expected as most of the SNPs selected for the array were chosen specifically because they segregate within *vinifera*. This observed pattern of MAF distributions across species also means that pairs of wild species are fixed for identical or alternative alleles at many SNPs across the genome, while comparisons between *vinifera* or *sylvestris* and any other wild species will tend to involve an intermediate frequency allele compared to an allele found at a frequency of either 0 or 1. We demonstrate this by showing that species pairwise comparisons involving *vinifera* or *sylvestris* exhibit many fewer SNPs that are fixed for the same allele compared to species pairwise comparisons not involving *vinifera* or *sylvestris* (Fig. 3a). One result of this is that *F_ST_* values from comparisons involving *V. vinifera* or *V. sylvestris* tend to generate intermediate *F_ST_* values since many SNPs are fixed within a wild *Vitis* species but segregate within *vinifera* or *sylvestris* (Fig. 3b). Moreover, the biased *F_ST_* values result in false phylogenetic inferences involving *vinifera* and *sylvestris* (described below).

### Genetic distances based on SNP genotypes

**Figure 4:**
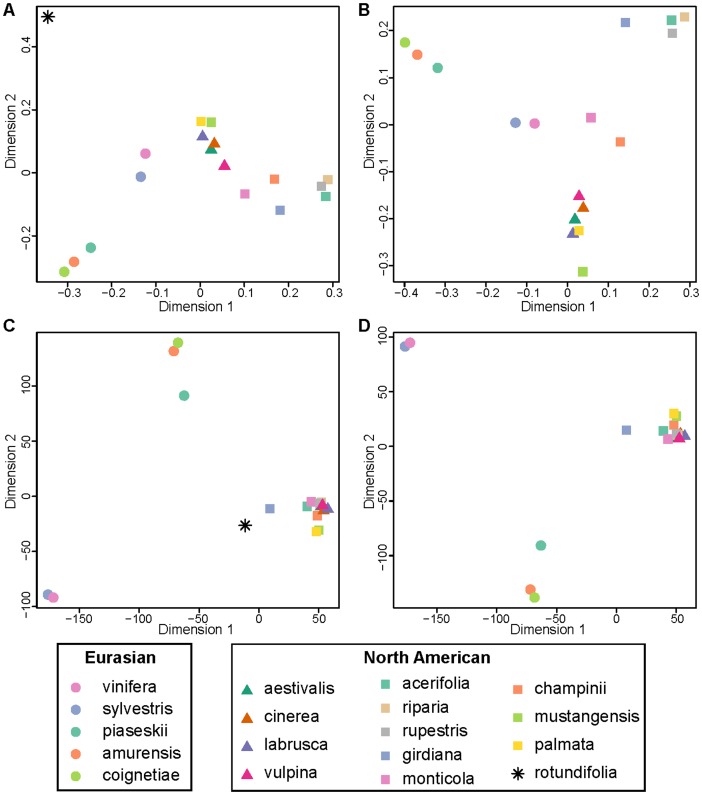
MDS plots of genetic distances among *Vitis* species using SNP genotype calls (A and B) and array hybridization intensities (C and D). A) MDS of *F_ST_* distances among all species calculated from genotype calls. B) Same as A) but without *rotundifolia*. C) MDS of genetic distances among all species based on intensity values. D) Same as C) but without *rotundifolia*.

**Figure 5:**
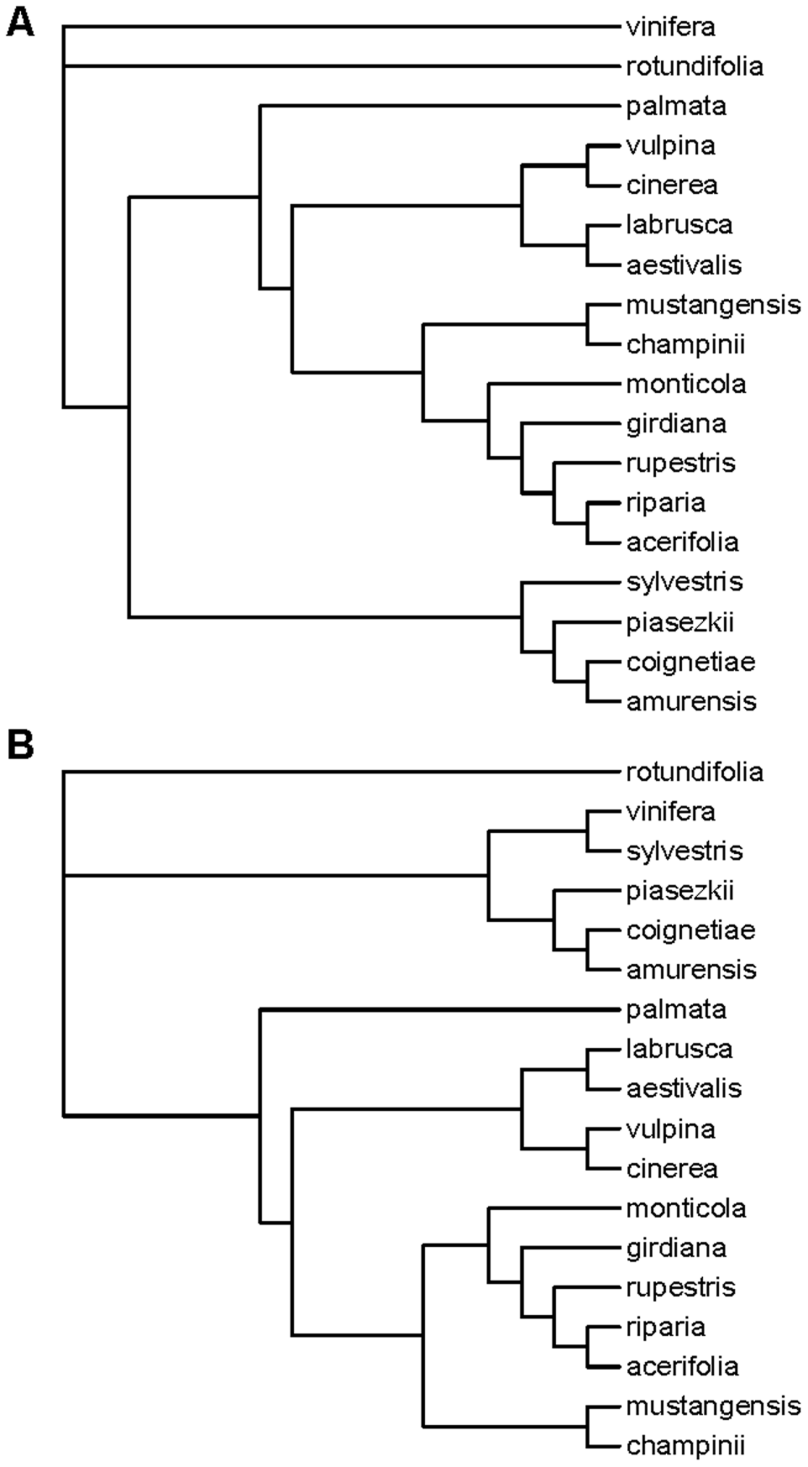
The phylogenetic tree of *Vitis* based on SNP genotype calls differs from the phylogenetic generated using array hybridization intensities. A) Neighbour-joining (NJ) tree from *F_ST_* estimates derived from SNP genotype calls from the Vitis9KSNP array. B) NJ tree from a distance measure derived from hybridization intensities from the Vitis9KSNP array.

Genetic distance among each pair of species was estimated using the *F_ST_* statistic and MDS plots were used to visualize the resulting genetic distances among all species. NJ trees were rooted with *V. rotundifolia* and completed for the full filtered dataset of 1030 samples. As was the case using PCA (Fig. 1), *V. rotundifolia* is clearly distantly related to other *Vitis* species based on *F_ST_* values (Figs. 4a, 5). However, *vinifera* and *sylvestris* appear misplaced in the MDS plot as they cluster more closely to North American *Vitis* than to Eurasian *Vitis* (Figs. 4a, b), which is neither in agreement with their geographic distribution nor with previous work [5]–[7]. Even more striking, phylogenetic analyses of the *F_ST_* distance matrix of SNP genotypes fail to group *vinifera* with *sylvestris*, a well-known progenitor-descendent pair (Fig. 5a). Phylogenetic analysis of the genotype data places *sylvestris* with other Eurasian species *V. amurensis*, *V. coignetieae*, and *V. piasezkii* while *vinifera* falls outside of a large clade of North American and Eurasian subgenus *Vitis*, alongside the sole representative of subgenus *Muscadinia*, *V. rotundifolia* (Fig. 5a). This placement of *vinifera* renders subgenus *Vitis* non-monophyletic based on SNP genotype calls and is inconsistent with all known evidence and previous work.

Despite the effect of ascertainment bias on inferring relationships to *vinifera*, the MDS (Figs. 4a–b) and phylogenetic analyses (Fig. 5a) of SNP genotype data resolve some relationships identified in previous *Vitis* analyses [5], [6]: 1) a Eurasian cluster in which *sylvestris* is basal to a group that includes *V. piasezkii* and *V. amurensis+V. coignetiae*; and 2) a clade of North American subgenus *Vitis* species in which *V. palmata* occupies the basal position; 2a) *V. aestivalis+V. labrusca* group together with *V. cinerea+V. vulpina*; and 2b) *V. champinii+V. mustangensis* form a clade that is sister to a clade of *(V. monticola (V. girdiana (V. rupestris (V. riparia+V. acerifolia))))*. With respect to Moore's classification scheme [68], these results support the monophyly of Moore's Series Ripariae (*V. acerifolia, V. riparia*, and *V. rupestris*), but do not support the monophyly of Series Cordifoliae (*V. monticola* Buckley, *V. palmata* Vahl, *V. vulpina* Linneaus), or Series Labruscae (*V. labrusca* Linneaus, *V. mustangensis* Buckley, *V. shuttleworthii* House). There is insufficient sampling/taxon identification (subspecific classification is not known for many accessions) to evaluate the monophyly of Moore's Series Aestivales (*V. aestivalis* Michaux, *V. aestivalis* var. *aestivalis*, *V. aestivalis* var. *bicolor* Dean, *V. aestivalis* var. *lincecumii* (Buckley) Munson) or Series Cinerescentes (*V. cinerea* Engelmann ex Millardet, *V. cinerea* var. *baileyana* (Munson) Comeaux, *V. cinerea* var *cinerea*, *V. cinerea* var. *floridana* Munson, *V. berlandieri* Planchon). SNP genotype data presented here corroborate several relationships identified in previous studies [5]–[7].

### Genetic distances based on hybridization intensities

The distance matrices generated from the various intensity data summary statistics (see Methods) were all highly correlated with one another (Mantel test; all pairwise comparisons p<1×10^−4^). This suggests that, regardless of the summary statistic used, the resulting genetic distance measures among species remain similar. Moreover, we compared phylogenetic tree topologies constructed from distance matrices derived from the various intensity data summary statistics and found that tree topology remains almost identical regardless of the summary statistic employed (Table S1). We therefore chose arbitrarily from among the summary statistics of the hybridization intensities and present results from the use of ln(X/Y). The genetic distance matrix generated from ln(X/Y) values was correlated with the *F_ST_* distance matrix (Mantel test, p = 0.021). However, the genetic distances derived from intensity values recover a more accurate phylogeny of the genus *Vitis* than the genetic distances calculated from SNP genotypes (Figs. 4c, d; 5b). Most notably, the intensity data-based phylogenetic analyses resolve *vinifera* and *sylvestris* as sister taxa which share a most recent common ancestor with the Eurasian clade of *V. piasezkii* and *V. amurensis+V. coignetiae*. This is consistent with other phylogenetic analyses of *Vitis* that have suggested a close relationship between the cultivated grape and Eurasian *Vitis* species [5]–[7].

Similar to the SNP genotype data, the intensity data resolve two clades within subgenus *Vitis*: 1) a Eurasian subgenus *Vitis* clade that includes (*sylvestris+vinifera*) and (*V. piasezkii* (*V. amurensis*+*V. coignetiae*)), and 2) a North American subgenus *Vitis* clade that includes *V. palmata* sister to 2a) (*V. labrusca+V. aestivalis) and (V. cinerea+V. vulpina*) and 2b) *V. monticola (V. girdiana (V. rupestris (V. acerifolia+V. riparia)))* and *(V. champinii+V. mustangensis)*. Similar to the SNP genotype analysis (described above), the intensity data support the monophyly of Moore's Series Ripariae and fail to support the monophyly of Series Cordifoliae and Series Labruscae. The monophyly of Series Aestivales and Series Cinerscentes cannot be evaluated given present sampling and lack of sub-specific taxon identification). Although the intensity data resolve the two main clades within subgenus *Vitis* (a clade of North American subgenus *Vitis* species and a clade of Eurasian subgenus *Vitis* species), they fail to resolve a monophyletic subgenus *Viti*s.

## Discussion

**Figure 6:**
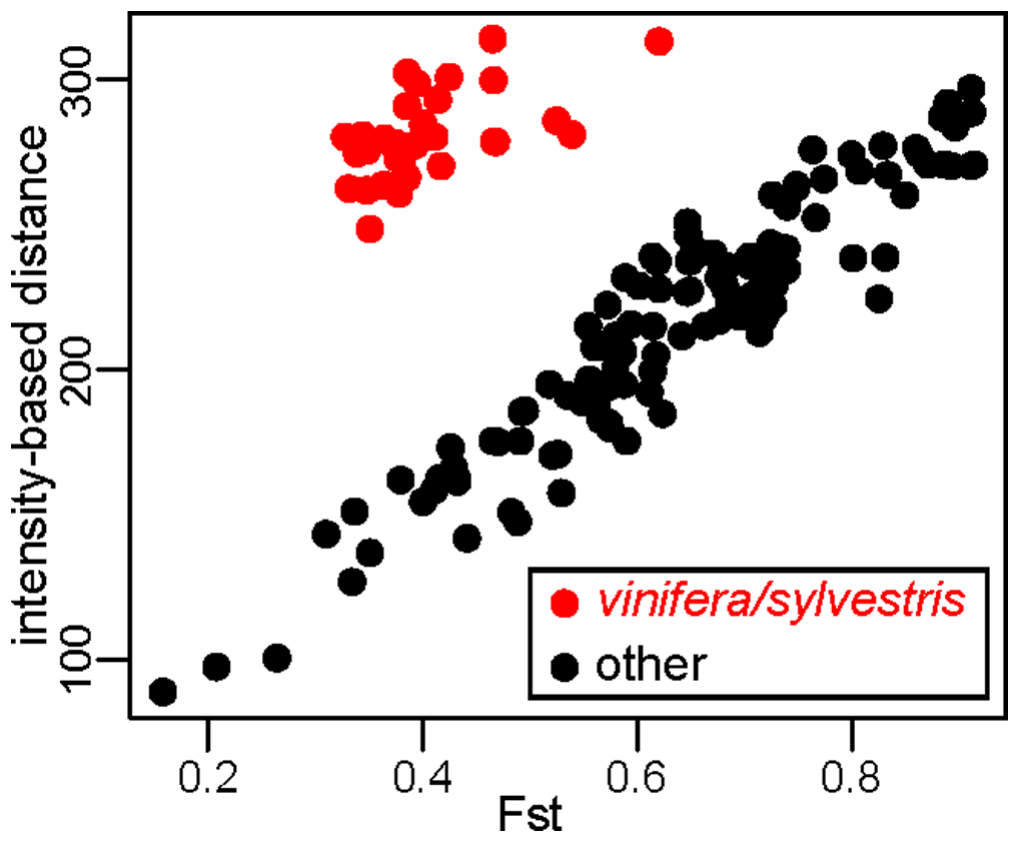
Comparison of genetic distance metrics based on genotype calls and hybridization intensities. Each dot represents a pairwise comparison between two species. Pairwise comparisons involving *V. vinifera* or *V. sylvestris* are highlighted in red. Genetic distances for *V. vinifera* and *V. sylvestris* based on intensity values are systematically elevated compared to other pairwise comparisons.

This study offers a phylogenomic approach to elucidating relationships in the North Temperate genus *Vitis*, which includes the most economically important berry species in the world, the cultivated grapevine *vinifera*. Leveraging a SNP array designed primarily for the cultivated grapevine, polymorphic sites discovered in *vinifera* and a small group of wild *Vitis* individuals were screened in over 1100 accessions representing 19 *Vitis* taxa, and used to reconstruct evolutionary relationships within *Vitis*. These data suggest that the Vitis9KSNP array suffers from ascertainment bias: SNPs were discovered mainly in *vinifera* and these SNPs are thus more likely to segregate in *vinifera* and its closely related ancestor *sylvestris* than in more distantly related wild *Vitis* species [14]. We investigated the effects of this ascertainment bias on phylogenetic inferences by analyzing relationships among diverse *Vitis* taxa using both SNP genotype calls and quantitative genotypes derived from hybridization intensity data. We demonstrate that ascertainment bias is pronounced when SNP genotypes are used to calculate genetic distances among taxa (Figs. 4a,b) and to construct phylogenies (Fig. 5a), leading to the failure to recover known clades. As an alternative to genotype calls plagued by ascertainment bias, summaries of hybridization intensity data provide a more accurate view of relationships among *Vitis* taxa (Figs. 4c,d; 5b). However, it is worth noting that even the hybridization intensity statistics are affected by ascertainment bias: genetic distance calculations based on intensity values involving *vinifera* or *sylvestris* are systematically upward biased (Fig. 6). This is unsurprising as we expect the probes on the Vitis9KSNP array, which were designed based on the Pinot Noir (*vinifera* cultivar) reference genome, to hybridize better to *vinifera* and *sylvestris* samples than to distantly related *Vitis* species whose sequences are not as complimentary to the probes on the array. Nevertheless, our analyses demonstrate that the severity of ascertainment bias when calling genotypes across diverse taxa results in incorrect phylogenetic inferences, while these obvious phylogenetic errors are not present when using intensity-based genetic distance measures. The data presented here confirm that SNP arrays developed for one taxon (e.g., *vinifera*) or one purpose (e.g., identifying gene regions associated with traits of agricultural importance) can be co-opted to study evolution and divergence at larger taxonomic scales, and that this work is enhanced significantly by the use of hybridization intensity data.

### Ascertainment bias in SNP arrays and the promise of hybridization intensity data in phylogenomics

SNP arrays have been developed for many crop plants [69] including apple [70], common bean [71]; citrus [51], corn [72], grape [14], peach [19], and rice [73], [74] for the purposes of population genetics, gene discovery, and marker-assisted selection. However, the application of these arrays to broader phylogenomic questions has been limited. Transferability of SNP arrays seems plausible in long-lived perennials that are particularly heterozygous [75]; for example [51], used SNPs recovered in the clementine genome to examine evolutionary relationships among over 50 diverse accessions in the complex *Citrus* genus. Data presented here provide further support for this, suggesting that SNP arrays have tremendous potential for expanding current understanding of evolutionary relationships among crop species and their wild relatives.

Ascertainment bias is known to interfere with population genetic inferences [76]. This study demonstrates that ascertainment bias is especially present in analyses above the species-level. The *Vitis* phylogeny built using SNP genotype data (Fig. 5a) failed to identify the close evolutionary link between the cultivated *vinifera* and its wild ancestor *sylvestris*, a well-known relationship that has been documented using molecular genetic data [5]–[7]. To address the problematic phylogeny that resulted from the ascertainment bias inherent in the genotype calls, we derived quantitative genotypes from the hybridization intensities and used these to estimate genetic distances among species. The resulting intensity-based phylogeny recovered most known clades and suggested other novel relationships not identified in previous analyses (described below).

### Implications of phylogenomic analyses for understanding evolutionary relationships within Vitis

Phylogenomic analyses of *Vitis* based on the Vitis9kSNP array data resolve several clades identified in previous analyses [5]–[7], [59]–[63] and suggest novel relationships not previously identified. On a broad phylogenetic scale, the hybridization intensity data support the distinction between subgenus *Muscadinia* (2n = 40) and two subgenus *Vitis* clades (*2n* = 38) [61]; however, neither the hybridization intensity analysis nor the SNP genotype analysis resolved a monophyletic subgenus *Vitis* (Fig. 5). This may be an example of ascertainment bias that is simply too strong to be overcome with hybridization intensity data. Of the 17 accessions used in the original discovery panel [14], only one came from subgenus *Muscadinia*. Perhaps any signal of differentiation between subgenus *Muscadinia* and subgenus *Vitis* may have been swamped by the sheer number of sites segregating within *vinifera*, and among *vinifera* and other subgenus *Vitis* taxa.

Subgenus *Vitis* exhibits a classic Eastern Asian-North American disjunct distribution with one species complex occurring in Eurasia. Although additional sampling representing both Eurasian and North American subgenus *Vitis* taxa is required to test the monophyly of the these groups, data presented here and in a previous study [60] indicate two evolutionarily distinct monophyletic groups within subgenus *Vitis*, one of which occupies Eurasia and the other which occupies North America. Some previous studies resolved a monophyletic Eurasian subgenus *Vitis* group, but did not support a monophyletic North American clade of subgenus *Vitis* [5], [7]. These studies suggested that North American *Vitis* species are ancestral within subgenus *Vitis*, and that a Eurasian subgenus *Vitis* group evolved from within the North American Subgenus *Vitis* clade. A different group of analyses reported a clade of North American subgenus *Vitis* species nested within a paraphyletic Asian subgenus *Vitis* [7], [63], and/or various degrees of intermixing among Eurasian and North American subgenus *Vitis* taxa [6], [60], [63]. The evolutionarily and geographically distinct Subgenus *Vitis* clades identified in this study could have resulted from a vicariant event (continental drift) leading to the geographic separation of Eurasian *Vitis* and North American *Vitis*, which was most likely associated by diversification of these groups on their respective continents [63]. A well-documented aspect of the North Temperate disjunct pattern is that genera displaying this geographic distribution generally have more Eurasian species than North American species possibly due to greater net speciation and rates of molecular evolution [77], [78]. This observation is corroborated in subgenus *Vitis*, where approximately 37 species have been recorded in Eurasia [58] and at least ∼17 taxa in North America (Moore and Wen, unpublished data).

North American subgenus *Vitis* species have been grouped by various authors, including M. O. Moore [68] who designated five series within subgenus *Vitis* in eastern North America based on morphological features: series Aestivales (includes *V. aestivalis*), series Cinerescentes (includes *V. cinerea*), series Cordifoliae (includes *V. monticola, V. palmata*, and *V. vulpina*), series Labruscae (includes *V. labrusca, V. mustangensis*, and *V. shuttleworthii*), and series Ripariae (includes *V. acerifolia, V. riparia*, and *V. rupestris*). Moore's (1991) [68] key to the series based on morphological features provides a framework of relationships among the series (Aestivales (Cinerscentes (Labruscae (Ripariae, Cordifoliae)))). Previous phylogenetic analyses have provided support for series Ripariae (*V. acerifolia, V. riparia*, and *V. rupestris*) [6], [7], [63]. Zecca et al. [5] resolved a clade with *V. riparia* and *V. rupestris*, but *V. acerifolia* grouped with *V. arizonica* and *V. girdiana*, among others. All analyses performed here support a sister-taxon relationship between *V. acerifolia* and *V. riparia*, which together form a clade with *V. rupestris*. Although a close relationship between *V. riparia* and *V. rupestris* is widely supported, discrepancy in the placement of *V. acerifolia* may indicate that this species has a hybrid origin derived from a cross between *V. riparia* or *V. rupestris* and one of the southwestern species.

Expanding upon the *V. acerifolia – riparia – rupestris* group, the hybridization intensity data provide evidence for a clade of subgenus *Vitis* species found primarily in the central-southern-southeastern United States (*V. riparia* is an exception to this) by placing the *V. acerifolia – riparia – rupestris* clade with *V. monticola, V. mustangensis*, and their hybrid derivative *V. x champinii* (*V. mustangensis* x *V. rupestris*) (Fig. 5). Like *V. acerifolia* and *V. rupestris*, *V. monticola* and *V. mustangensis* are species whose primary distributions are in the central to central-southern United States. *Vitis riparia* is a widespread climbing vine found throughout the Midwest and the northeastern quarter of the United States. Previous authors grouped *V. monticola* with *V. palmata* and *V. vulpina* based on morphology [68], but some recent molecular analyses have suggested a relationship between *V. monticola*, *V. mustangensis* and the *V. acerifolia – riparia – rupestris* group [6] but see [7]. The SNP genotype calls place the Californian species *V. girdiana* in this group as well, consistent with previous analyses [5]–[7].

A second major clade within North American subgenus *Vitis* includes two species pairs: *V. aestivalis*+*V. labrusca* and *V. cinerea*+*V. vulpina*; *V. palmata* is basal among all North American subgenus *Vitis* species. *Vitis aestivalis, V. cinerea, V. labrusca*, and *V. vulpina* have largely overlapping distributions in the eastern half of the United States. These species clustered together in earlier studies [5]; most recently [7], identified a clade of (*V aestivalis+V. labrusca*)+*V. vulpina*, and a second clade of ([*V. cinerea*+*V. palmata*)+(*V. mustangensis*+*V. shuttleworthii*)]. While both this study and [7] find support for a close relationship between *V. aestivalis* and *V. labrusca*, the positions of *V. monticola*, *V. palmata*, and *V. vulpina* differ in the two analyses. The results of both studies conflict with Moore's [68], [79] classification scheme. For example, Moore's series Cordifoliae includes *V. monticola, V. palmata*, and *V. vulpina*; analyses presented here suggest *V. palmata* is basal in North American subgenus *Vitis* and that *V. vulpina* forms a clade with *V. cinerea*. Similarly, Moore's [68] series Labrusceae posits a close relationship between *V. labrusca, V. mustangensis*, and *V. shuttleworthii* (not sampled in this study). However, data presented here suggest *V. labrusca* forms a clade with *V. aestivalis*, and that *V. mustangensis* groups with *V. acerifolia, V. monticola, V. riparia*, and *V. rupestris*.

Phylogenetic relationships of crop wild relatives can provide insights into the evolutionary history of a crop as well as a window into contemporary evolutionary processes such as hybridization between cultivated populations and wild progenitors or processes driving divergence among closely related species (e.g., [61]). In the case of grape, the wild progenitor and geographic origins of domesticated European grapevine are well known [55], [57], [79]. However, lesser-known components of grapevine evolutionary biology include relationships among species that are used as parents in hybrid crosses (e.g., *V. aestivalis*, *V. labrusca, V. vinifera*) or those that are used as rootstocks (e.g., *V. cinerea* var. *helleri, V. riparia*, *V. rupestris*). For example, grafting *vinifera* scions to rootstocks of non-*vinifera* species dates back to the mid-1900's when the phylloxera invasion of France threatened to destroy the French grape crop [80]. Rootstocks used to support *vinifera* come almost exclusively from North American species [81]. Recently, hybrids between *V. cinerea* var. *helleri* and *V. riparia* or *V. rupestris* have been used to produce rootstock that is easy to propagate and that can withstand challenging abiotic conditions [80]. Data presented here demonstrate that these important rootstock species occur in different clades within the North American subgenus *Vitis: V. cinerea* is most closely related to *V. vulpina*, while *V. riparia* and *V. rupestris* form a clade together with *V. acerifolia*. Building upon this phylogenetic framework, future work characterizing the diversity of abiotic and biotic pressures faced by natural populations and the genetic basis of abiotic and biotic stress response, will expand understanding of evolution and adaptation in *Vitis*, and may provide molecular tools to facilitate marker-assisted selection for rootstocks.

## Conclusions

This study demonstrates that ascertainment bias presents a significant challenge for the application of SNP arrays in phylogenetic reconstruction; however, the effects of ascertainment bias can be minimized by using hybridization intensity rather than SNP genotype calls. We demonstrate that the Vitis9kSNP array, a panel developed based on variation discovered in 11 accessions of *vinifera* and single accessions of six other *Vitis* species, can be used to screen variation in a broad sample of over 1100 samples representing 18 taxa. Resulting data confirm relationships identified in previous studies (e.g., *V. riparia+V. rupestris*, *vinifera*+*sylvestris*) and suggest novel affinities among taxa (e.g., *V. aestivalis*+*V. labrusca* and *V. cinerea+V. vulpina*). This phylogenomic analysis of *Vitis* demonstrates the utility of SNP arrays in phylogeny reconstruction and expands current understanding of relationships among North American subgenus *Vitis* species.

## Supporting Information

Table S1 **Comparison of the performance of various genetic distance measures based on hybridization intensity.** The values within each cell represent a measure of the difference between tree topologies generated from the summary statistics found in the respective row and column names. The distance measure between the pair of phylogenetic trees is defined as the twice the number of internal branches defining different bipartitions of the tips (Penny and Hendy 1985). All of the summary statistics generated phylogenetic trees with highly similar topologies.
(DOCX)
