# Correction: *Vitis* Phylogenomics: Hybridization Intensities from a SNP Array Outperform Genotype Calls

**DOI:** 10.1371/annotation/283e5eba-a65d-42a3-a430-6ca0be86147c

**Published:** 2014-01-06

**Authors:** Allison J. Miller, Naim Matasci, Heidi Schwaninger, Mallikarjuna K. Aradhya, Bernard Prins, Gan-Yuan Zhong, Charles Simon, Edward S. Buckler, Sean Myles

An additional affiliation for the eighth author was incorrectly omitted from the byline. In addition to institution number 6, Edward S. Buckler is also affiliated with the following institution: USA Department of Agriculture (USDA) - Agricultural Research Service (USDA-ARS).

