# Supplementary material for: Vitis Phylogenomics: Hybridization Intensities from a SNP Array Outperform Genotype Calls
Source: PLoS One. 2013 Nov 13;8(11):e78680. doi: 10.1371/journal.pone.0078680 (PMC3827278; doi:10.1371/journal.pone.0078680)
Supplement: Table S1 — Comparison of the performance of various genetic distance measures based on hybridization intensity. The values within each cell represent a measure of the difference between tree topologies generated from the summary statistics found in the respective row and column names. The distance measure between the pair of phylogenetic trees is defined as the twice the number of internal branches defining different bipartitions of the tips (Penny and Hendy 1985). All of the summary statistics generated phylogenetic trees with highly similar topologies. (DOCX) [file pone.0078680.s001.docx]

**Table S1:** Comparison of the performance of various genetic distance measures based on hybridization intensity. The values within each cell represent a measure of the difference between tree topologies generated from the summary statistics found in the respective row and column names. The distance measure between the pair of phylogenetic trees is defined as the twice the number of internal branches defining different bipartitions of the tips (Penny and Hendy 1985). All of the summary statistics generated phylogenetic trees with highly similar topologies.

|  | **X** | **Y** | **X+Y** | **ln(Y/(X+Y))** | **ln(X/(X+Y))** | **ln(X/Y)** |
| --- | --- | --- | --- | --- | --- | --- |
| **X** | 0 |  |  |  |  |  |
| **Y** | 0 | 0 |  |  |  |  |
| **X+Y** | 6 | 6 | 0 |  |  |  |
| **ln(Y/(X+Y))** | 2 | 2 | 6 | 0 |  |  |
| **ln(X/(X+Y))** | 0 | 0 | 6 | 2 | 0 |  |
| **ln(X/Y)** | 0 | 0 | 6 | 2 | 0 | 0 |
